# Emergency management in health: key issues and challenges in the UK

**DOI:** 10.1186/1471-2458-12-884

**Published:** 2012-10-19

**Authors:** Andrew CK Lee, Wendy Phillips, Kirsty Challen, Steve Goodacre

**Affiliations:** 1 School of Health and Related Research, The University of Sheffield, Sheffield, UK

**Keywords:** Emergency management, Emergency planning, Emergency preparedness, Disaster planning, Disaster management

## Abstract

**Background:**

Emergency planning in the UK has grown considerably in recent years, galvanised by the threat of terrorism. However, deficiencies in NHS emergency planning were identified and the evidence-base that underpins it is questionable. Inconsistencies in terminologies and concepts also exist. Different models of emergency management exist internationally but the optimal system is unknown. This study examines the evidence-base and evidence requirements for emergency planning in the UK health context.

**Methods:**

The study involved semi-structured interviews with key stakeholders and opinion leaders. Purposive sampling was used to obtain a breadth of views from various agencies involved in emergency planning and response. Interviews were then analysed using a grounded approach using standard framework analysis techniques.

**Results:**

We conducted 17 key informant interviews. Interviewees identified greater gaps in operational than technical aspects of emergency planning. Social and behavioural knowledge gaps were highlighted with regards to how individuals and organisations deal with risk and behave in emergencies. Evidence-based approaches to public engagement and for developing community resilience to disasters are lacking. Other gaps included how knowledge was developed and used. Conflicting views with regards to the optimal configuration and operation of the emergency management system were voiced.

**Conclusions:**

Four thematic categories for future research emerged:

(i) Knowledge-base for emergency management: Further exploration is needed of how knowledge is acquired, valued, disseminated, adopted and retained.

(ii) Social and behavioural issues: Greater understanding of how individuals approach risk and behave in emergencies is required.

(iii) Organisational issues in emergencies: Several conflicting organisational issues were identified; value of planning versus plans, flexible versus standardized procedures, top-down versus bottom-up engagement, generic versus specific planning, and reactive versus proactive approaches to emergencies.

(iv) Emergency management system: More study is required of system-wide issues relating to system configuration and operation, public engagement, and how emergency planning is assessed.

## Background

The UK experiences around 11 major incidents per year [1]. These often require coordinated multi-agency responses including from the National Health Service (NHS). Previously, this work was conducted in the background but the horrifying terrorist attacks of 11 September 2001 and 7 July 2005 have catapulted the emergency management field up the political agenda [2]. Since then, research and publications in this field have accelerated, as demonstrated by the US experience following 11 September 2011 where in the past decade nearly seven hundred articles were published pertaining to this single event alone [3].

**Figure 1:**
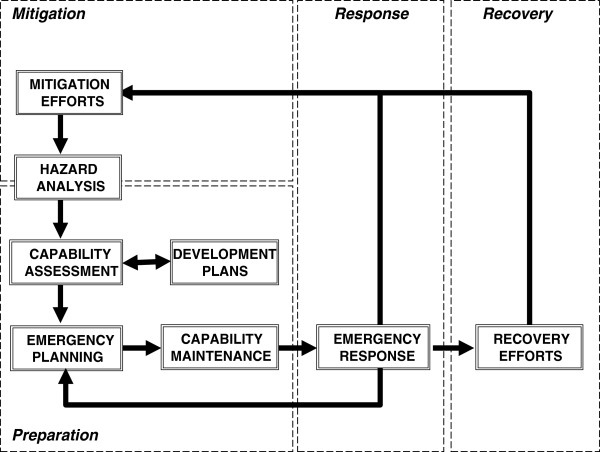
**The emergency management cycle.** This figure illustrates the key stages of emergency management from mitigation, through to preparedness (covering emergency planning, capability assessment and maintenance), emergency response and eventually recovery. This is derived from the work of McLoughlin (1985) who detailed the various stages and how they are related in an ‘integrated emergency management system’.

Emergency management is often erroneously understood as only those activities pertaining to the response to an emergency situation. In its broadest sense however, it is synonymous with emergency planning and encompasses a spectrum of activities from business continuity management and planning, training and preparedness, as well as the response to, and recovery from emergencies. (Figure 1) [4,5] This was further codified in the Civil Contingencies Act, 2004 that set out for the various health agencies key responsibilities to prepare for major incidents that included the assessment of local hazards and risks, planning, training and testing activities [6,7]. 

However, deficiencies in NHS emergency planning have been previously noted [3-6], and questions have been raised as to the evidence-base that underpins much of the activity of emergency planning for major incidents [8]. Also lacking is an evidence-base to support planning around longer-term “rising tide” incidents such as infectious disease outbreaks, covert chemical, biological, radiological, and nuclear events, and threats to infrastructure, and business continuity, such as floods and transport strikes. There are also inconsistencies in terminologies and concepts used [9,10]. Different models of emergency management exist worldwide that reflect the situational contexts of the countries in which the systems have evolved [6,11,12]. What is less clear is what systems and processes work best.

We present the results from a study commissioned by the National Institute for Health Research Service Development Organisation to determine the evidence-base for emergency planning, specifically for the UK health context [13]. This project was a collaborative partnership between academics, clinicians, public health and health protection specialists. It consisted of 4 subprojects: a scoping study of the published literature, a qualitative grey literature scoping review, key informant interviews, and an e-Delphi study. Mixed methods were employed in order to approach the topic holistically. We report here the key issues and challenges for emergency planning in health in the UK that were identified.

## Methods

We sought to gather more detailed insight into the state of emergency planning in health in the UK and any research gaps. In order to do so, we chose a qualitative approach, using semi-structured interviews with key stakeholders and opinion leaders acting as our key informants.

Ethics approval for this study was sought and received from the NHS Sheffield Research Ethics Committee **(REC Ref 10/H1308/67).**

The sampling strategy adopted was purposive in order to obtain a breadth of views from multi-agency stakeholders. Initially, we identified a list of potential key informants to interview. These individuals were selected on the basis of their known expertise, recognized experience, or research in the field. They were identified through the World Association for Disaster & Emergency Medicine, the Department of Health Emergency Planning Clinical Leaders Advisory Group, faculty of the Masters course in Health Incident Command at Manchester Metropolitan University, senior faculty of the Emergency Planning College and the Health Protection Agency (HPA). Public representation was also sought through the Sheffield Emergency Care Forum, a group of interested members of the public who are involved in providing a public voice, interest or lay representation in research in Sheffield.

Potential participants were initially contacted by telephone, e-mail, and/or letter, with summary information on the project. Those participants who agreed to take part in the project were provided with participant information leaflets and returned a signed consent form or correspondence agreeing to participate. A mutually agreed date, time and venue were then set for the interviews. The interviews were carried out either face-to-face or by telephone depending on the wishes of the respondents.

Of 50 potential key informants identified, twenty-seven were approached and invited to interview. Of this number, 17 key informants agreed to be interviewed. The reason for non-participation by key informants invited who decline interview is not known as this information was not collected. The participants included a range of individuals who in their professional capacities included emergency planners, health managers, policymakers, technical experts and scientific advisors. (Table 1) There was representation from the public, private sector, the military, primary and secondary care, ambulance service, civil service, and the HPA. Some interviewees operated at the frontline locally, whilst others participated at more senior levels in government as well as internationally. We are confident that a broad and appropriate range of informants were included and covered.

**Table 1:** Profiles of the key informants interviewed

**Interviewee**	**Practitioner**	**Technical expert**	**Scientific or academic expert**	**Policymaker**	**Member of the public**
A			√		
B					√
C	√	√			
D					√
E					√
F	√	√			
G			√		
H		√			
I				√	
J				√	
K	√	√			
L	√	√	√		
M	√	√		√	
N		√	√		
O	√				
P	√				
Q	√				

The interview schedule was developed from our preliminary conceptual mapping and scoping of the literature done as part of the wider study [13]. The schedule consisted of several broad themes to be explored that included those themes we had identified as potential issues. An iterative approach was adopted and the interview guide was modified over the course of the project to explore emergent themes that had not been identified *a priori*.

Interviews were carried out initially by two researchers together to standardise the interview process and for familiarisation with the process. Subsequent interviews were conducted by the researchers individually. Both researchers were dually experienced as academics as well as in the field of emergency planning. This was considered important as it would help facilitate discussions that could be technical in nature. The researchers were mindful throughout of the potential for observer bias in view of their previous expertise and experience in this field and how this could influence their interpretation of findings. To mitigate this, periodic discussions between the researchers were held to compare and contrast findings.

Most of the interviews were carried out face-to-face either at the participants’ workplace or at a university venue to suit the participants’ convenience. A small proportion of interviews were conducted over the telephone as agreed with the interviewees in advance. Interviews were recorded using digital audio recorders and transcribed verbatim with the participant’s informed consent. Concurrent notes were also made during the course of the interviews. Quotes were anonymised to protect the identities of participants.

Data from interviews were then analysed using a mix of thematic analysis to explore and describe issues and themes, as well as using a constant comparison grounded approach to try and identify a conceptual framework. After familiarisation with the material, coding was undertaken. This utilised several variants of coding that included descriptive coding, in vivo coding, and versus coding approaches [14]. We were especially interested in trying to identify contrasting views from respondents or issues raised where there were tensions or uncertainty. The codes were then categorized and amalgamated into higher level thematic categories, and re-iterated as new codes where appropriate. These were then mapped out to display their linkages.

## Results

**Figure 2:**
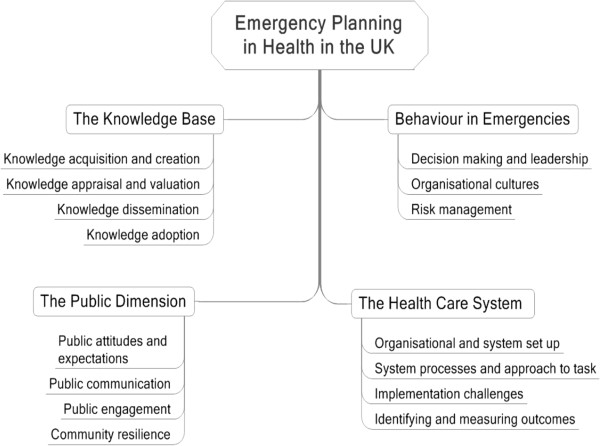
**Summary of thematic categories identified.** The key thematic categories identified include the knowledge-base used for emergency management, individual and organisational behaviour, health care system issues and matters relating to the public in crisis/disaster situations. This figure further maps out the major themes linked with each category. Under the knowledge-base, it includes issues of how knowledge is acquired, appraised, disseminated, adopted and retained. The category of behaviour in emergencies includes decision-making, organisational behaviour in crisis as well as risk management. Health care system issues cover organisational set up and configuration of the emergency management system, process issues of how the system operates, implementation challenges, as well as problems with how outcomes are identified and measured in emergencies. The final category, the public dimension, covers public attitudes and expectations, communication with the public, public engagement and the development of community resilience.

From the key informant interviews we identified 4 emergent thematic categories relevant to emergency planning in health (Figure 2):

(i) the knowledge (or evidence-) base for emergency planning,

(ii) how individuals and organisations react and behave in emergencies,

(iii) the healthcare system in which the emergency management occurs, and

(iv) issues related to the public served by the system.

### The knowledge base

Many issues were raised pertaining to various aspects of the knowledge base for emergency management. Firstly, there was an issue of how knowledge was acquired. Unlike traditional biomedical science where knowledge is built up from research, this was not the case for emergency management. Due to the unpredictability of emergencies, and the inherent slowness in current research commissioning processes, the status quo does not facilitate the accumulation of research-based knowledge.

“It’s very difficult to, you can’t do a randomised control trial. You can’t compare because every situation is very different.”

(Emergency Planning Technical Expert 1)

“Emergency planning is an unusual area … If you do sort of medical research you test your hypothesis and then you sort of devise the treatment or devise a drug and you test it etc. Emergency planning is nothing like that at all.”

(Emergency Planning Policymaker 2)

“Those quick response reports (research in the US) are then published online for people to see, so you can see what is happening and there is this much more “joined up” thinking between government and research and the practitioner in the United States. National Science Foundation funds an awful lot of work in disasters and emergency management, which we do not do here.”

(Emergency Planning Academic and Technical Expert 1)

There were inter-professional differences in how the existing knowledge is viewed, valued and used, as well as the “appetite” for evidence. “Blue light” emergency services practitioners equated experience of dealing with incidents with expertise and as evidence. In contrast, stakeholders from a health background (e.g. medicine and nursing) valued “knowledge” that had been peer-reviewed and published.

“You’ve got with a lot of practitioners this brick wall that you have to kick them through so that they can open their eyes you know. It’s very much ‘Why do I need to know that? What’s that gonna help me? Why should I read a book about the way disasters may happen or about social vulnerability? What will that do for me in terms of helping respond to an emergency?’”

(Emergency Planning Academic and Technical Expert 1)

“Well a lot of people (emergency planners and managers) don’t see the relevance (of evidence) or how it can be done.”

(Emergency Planning Technical Expert 2)

“…the difference in cultures and the like and the knowledge and evidence it comes out of the culture aspects of how (the different organisations) do it. Some are sort of disorganised … This is part of the problem I’ve noticed in the exercises we’ve had as to what each see as the evidence they need and how they approach it.”

(Public representative 3)

There were also differences in how the knowledge was valued; the former valued practical “knowledge” more than academic literature for example. The degree to which information was scrutinized also differed. Emergency planning practitioners tended to be less critical of their information sources, accepting them at ‘face value’.

“People talk about stuff as if this is the way we did it and therefore it’s right when that is simply anecdotes or based on experience. It hasn’t been evaluated independently and found to be something that is applicable universally in other situations … The evidence base is only anecdotal and perhaps that’s symptomatic of the field itself … To me it reflects the fact that what people take as quality assured knowledge is different from what they may just glean from all sorts of different sources. The question is how far they actually can judge what is good quality information and procedures and what is just what they have picked up from somewhere else …”

(Emergency Planning Academic and Technical Expert 2)

This raises the issue of not just how practitioners acquire knowledge, but also how they may sufficiently discern its credibility and value. Consequently, the use of evidence in emergency planning, as one respondent describes, is “often patchy and impoverished”.

There were also issues with how knowledge was transferred from academia to practitioners, how it is cascaded within organisations and communicated between organisations. Problems with these ‘knowledge transactions’ often hinder the dissemination of knowledge. In turn, the knowledge needed to be adopted and implemented, as well as retained within organisations, and again problems were reported. This also includes issues with how knowledge is contextualised and occasionally mis-applied to local situations.

“We don’t share our research with our practitioners in a good way. … (We need to) develop that mechanism for knowledge exchange … It’s getting the knowledge out there about what happens, why it happens, making people aware.”

(Emergency Planning Academic and Technical Expert 1)

“A lot of people do research in this area and then are very frustrated that they don’t get their research into policy practice because they actually don’t share it with the people who need to know what’s there because they don’t know how to share it.”

(Emergency Planning Technical Expert and Policymaker)

 Another concern was the difficulty of maintaining organisational memory of lessons learnt from previous incidents. The need for an easily accessible repository of knowledge was identified which is currently lacking.

“… in wanting to get messages across or work with people, you know when you are working with different kinds of people, like in the academic and the practitioner world, you have to move over to their world. Think of the world through their eyes and communicate in the way that they will hear and if you can’t do that, then no matter how good your research is, it is not relevant to them … The thing that worries me most at the moment is having a corporate memory … a way of capturing knowledge, sharing knowledge … That is one thing that we are rapidly losing … knowledge and understanding and people re-inventing wheels. They say we haven't done this before and you say you have, you just don't know about it.”

(Emergency Planning Academic and Technical Expert 2)

“… these settings are so far and few between and therefore lessons learnt are often forgotten until the next time …”

(Health Technical Expert)

### Behaviour in emergencies

Most respondents were less troubled with technical knowledge gaps, but more concerned with social and behavioural science gaps. There is a lack of understanding of how individuals (both the public and providers) behave in emergencies. Neither is it known what constitutes good decision-making or good leadership in an emergency.

“I would give (research funding priority) to the social scientists … to understand a bit more of why in a crisis individuals and organisations behave as they do. To get under the skin of what goes well and what doesn’t go well in emergency health response … Something a bit deeper, something that can you know, tackle issues of power, implied power, command, control and locus of control and kind of stuff that maybe a kind of a hard, hard nosed physical scientist would say is all a bit woolly but in actual fact I think it’s probably quite fundamental to this.”

(Scientific and Technical Expert)

“(The big gaps are) around behavioural sciences because I think when we do the major exercises there’s often the lack of understanding of how people actually react in emergencies or incidents … We really don’t understand how the public will react to (a disaster) if it happens.”

(Emergency Planning Technical Expert 2)

Closely associated with individual and organisational behaviours are the different organisational cultures that exist and the manifestations of inter-professional culture clashes. There are marked variations in the different agencies’ understanding of risks, the situation, how they communicate, react, respond to, recover and learn from incidents.

It was also observed that the current approach to emergencies in the UK tends to be reactive, less pre-planned, with decision-makers tending to “muddle through” situations until their eventual resolution. It is overly focussed on the “response” phase with less attention paid to other aspects such as mitigation and recovery. It was also noted that practitioners tended to make assumptions that feed into the emergency planning process. This introduces potential vulnerabilities into emergency plans, preparations and subsequent responses.

“We do sometimes tend to say we’re good at this. We’ve done so many but we’re good at all this and I’m not so sure we are … You’ve got to be very honest with yourself and be very careful that you don’t over-estimate your own knowledge and your own skills. Just because you’ve dealt with this in the past does not necessarily mean that you’ve done the right thing. It may just mean that you’ve got away with it!”

(Health Technical Expert and Policymaker)

“I don’t think there was a plan and certainly it wasn’t kind of thought through and implemented that systematically. It seemed to me there were plenty of resources thrown at the problem but (the response) happened despite not having a plan rather than because there was a plan.”

(Scientific and Technical Expert)

“We make a lot of our planning here based on a lot of assumptions which really haven’t been thought through.”

(Emergency Planning Academic and Technical Expert 1)

Of special note was how risks are perceived differently by individuals and agencies. The occupational backgrounds of the individuals involved in particular influenced risk perception. There was also conflicting and varying “sensitivity” to risk; some agencies were better prepared for known threats than the unexpected ones, whereas others paradoxically became less reactive to familiar threats.

“I think to that we have possibly focused a little bit on the wrong end of things and very much on the "Big bang" high profile emergencies and less on the less, the sort of "slow burn" or things like winter preparedness, planning for winter… I think the fact that many emergency planners were recruited from the Forces and they … tended to be driven by "Big bang", what I call "Big bang" events, and therefore they tend to plan for "Big bang" events, whereas the sort of events that might really bring the health service down like a very bad winter or a very bad outbreak … or a combination of that and IT failure were less likely to be planned for.”

(Emergency Planning Academic and Technical Expert 1)

Another problem was how risk is communicated to other agencies, policymakers and the public, as well as how the different agencies respond to risk. Health organisations were more risk averse and tended to “play safe” and “over-react” to threats compared to the other emergency responders.

“The trouble is around risk assessment, the perception or risks and understanding of risks. There’s a massive role for academia there. There’s language around risk, there’s a language around perception of risk and communication of risk that we need to be able to bring in more to help the general public understand what they’re faced with and how they see risk and how we communicate risk.”

(Emergency Planning Policymaker 2)

### Health care system issues

Several themes related to systems-level issues were also identified. Different emergency management systems exist worldwide and it is not known how well the different systems perform relative to one another. Neither was there a way of gauging the performance of each system. What is clear is that there are socio-political contextual specificities that influence the organisation, function and effectiveness of the various emergency management systems.

“I’ve been to Australia and Holland looking at some of the stuff that they do on emergency planning and the cultures are so different in terms of the expectations of the population, of the public services, the extent to which the public are involved in planning and you know the split of responsibilities between the individuals and the community themselves and the public sector is often so different that it’s quite difficult. The political systems are often so different.”

(Emergency Planning Technical Expert 1)

“We tend to see the science base as evidence base says X therefore X is right. But actually X may not be right in different circumstances because the evidence base was developed in different circumstances. And we need to be a bit more flexible and also to recognise that you know if you make a decision in the absence of information your decision may be different when you get the information.”

(Emergency Planning Technical Expert 2)

In the UK, it was also observed that there is a lack of understanding of the role of the health service by the health service in emergency planning. Much of it tended to be focused on operational response aspects, and there is a perceived lack of a strategic “whole systems approach” to emergencies. Recovery issues in particular tended to be neglected.

“The NHS is the last organisation to “switch on” … The NHS deals poorly with uncertainty.”

(Health Emergency Planning Manager 3)

“Everybody is now so risk averse that everything’s got to be so detailed in its planning that actually we’re moving towards a hiatus. The senior directors of all sorts of companies and organisations are then feeling hamstrung or blinded by ‘Get me a plan on X, Y and Z” ‘cos this is what’s happened. But actually it’s not X, Y, Z that’s happened; it’s something that falls in-between.”

(Emergency Planning Policymaker 1)

There were tensions and conflicting views as to the right approach to emergency management. Is it better to have generic or specific plans, a flexible approach to a situation or strict adherence to plans and pre-set measures, or, a top-down or bottom-up approach to the conduct of the response? There was also a disconnect between those who wrote plans and those who implemented them which consequently meant that what was planned did not always match what was eventually implemented.

“My own personal experience of the pandemic are that plans are nothing, planning is everything. So did I once refer to the DH pandemic plan during my roles in the pandemic? No. Did I once ever see that document out on a table during the pandemic itself? No. Do I think that matters? No, I don’t because I think the plan was a living embodiment of the fact that the planning had taken place…”

(Scientific and Technical Expert)

“I think you need to have a system that’s flexible, but how flexible?”

(Health Technical Expert and Policymaker)

“I think the only way to manage a large incident has to be SOPs, protocol-based as to what you were going to do and of course for a lot of this you are going to be doing things that you wouldn’t normally do you know…So I think, I would tend to be in the inflexible group. I think the only way you can manage a big incident is by having very rigid protocols and driving that forward… I think one has to be absolutely rigid to manage this in everyone’s best interest.”

(Health Technical Expert)

“It’s probably difficult to square in a sort of non-military role but how do you get people who are leaders into a job that’s not always dealing with conflict, has to mix between … You’ve got to crack on and put some form of command and control in for this particular moment in time but actually the majority of your time’s spent cajoling, encouraging and taking on board stakeholder agreement and consensus … getting there eventually but not quite in the fashion that you want it.”

(Emergency Planning Policymaker 1)

Furthermore, whilst an integrated response is desired, in reality the various organisations involved usually operate independently in silos and the wider health system response is insufficiently integrated. Civil society organisations are often marginalised as well. Health organisations tended to be slow and resistant to change. In addition, they were less familiar with dealing with emergency situations and were more focused on their routine workload.

### Public issues

It was felt that the public in the UK did not understand emergency planning and opportunities for the public to engage in emergency planning were limited. There was a perception that the public expected an external agency to protect them and provide for them in emergencies.

“The public really want to be sure that things are happening and they are going to be sorted out and looked after and made safe if anything goes wrong.”

(Public representative 3)

Some interviewees described this as a “culture of dependency” and there was a shared concern that there are unrealistic public expectations of what the emergency services can do in an emergency. Although greater shared decision-making and public engagement seems to be sought, the stakeholders involved did not appear to know how best to achieve this. Similarly, whilst there was a lot of discussion of the concept of community resilience and an understanding that public engagement was key, there was again a lack of clarity as to what “meaningful engagement” entailed.

“I think the most consistent lessons learned from all these things is about failure to communicate both between responding agencies and with the public, and one would have to assume that if that keeps recurring as a regular theme in lessons learnt then there is probably a question in there that hasn’t been properly answered.”

(Emergency Planning Technical Expert 2)

“I think we are moving towards a culture of more of an understanding of human behaviour and trusting the public and all of that. But that’s filtered through political priorities and you know the facilities around sharing information and sharing decision-making and the political interpretation…”

(Emergency Planning Academic and Technical Expert 2)

“Getting the public involved with some of these processes would be a good thing to do. In the absence of a science base … at least we would have a different perspective on the problem rather than the assumptions made by experts and planners.”

(Emergency Planning Technical Expert 2)

“Engagement is difficult. (If) there is no obvious way how you can influence (things), you don’t get involved and because we don’t get involved you know it’s kind of a vicious circle and by changing that and making it into a virtuous circle potentially you could begin to help change that.”

(Emergency Planning Technical Expert 1)

## Discussion

### Summary of study findings

**Figure 3:**
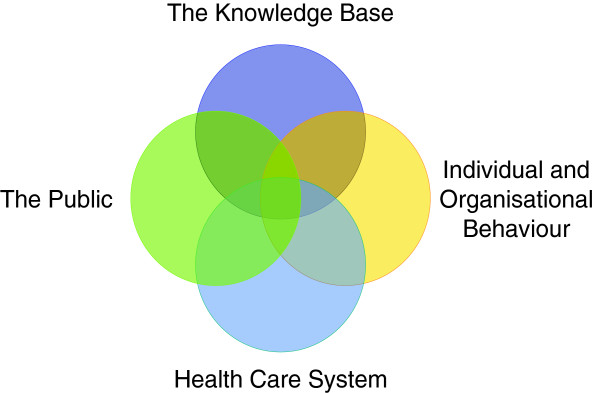
**Key thematic categories identified.** The key themes identified by the study group naturally into 4 distinct thematic categories: the knowledge-base used for emergency management, individual and organisational behaviour, health care system issues and matters relating to the public in crisis/disaster situations. These thematic categories are not independent but show considerable overlap as illustrated in this Venn diagram.

Four interconnected thematic domains were identified around the knowledge-base for emergency planning, how individuals and organisations react and behave in emergencies, the health care system in which the emergency management occurs, and the public served by the system. (Figure 3) For example, decision-making by emergency managers was to some extent based on the available evidence-base, but heavily influenced by the individuals’ experiences and professional backgrounds. This in turn was affected by the organisational cultures in which the individuals worked in and the set up of the healthcare system. The decisions subsequently impact on the intended beneficiary, the public. However, the public is not a static entity but a dynamic living community of individuals who themselves perceive and react to the socio-political environment [15]. Emergency management therefore cannot be seen in isolation in its individual components, but should ideally be addressed holistically at a systems level. 

### What is already known

There is a considerable body of literature and ongoing research worldwide on emergency management from both developed and developing countries [13,16]. Much of it is from North America pertaining particularly to emergency preparedness and response, and on hazard-specific topics such as countermeasures to terrorism, flood warning systems and earthquake prediction, or the application of technologies such as geographical information systems. Literature on organisational issues such as incident command system set-up however is uncommon. Neither is there much on knowledge management and learning from disasters, or performance management including the measurement of effectiveness or efficiency of response. A better understanding of how individuals approach risk and behave in emergencies, as well as means of strengthening community resilience have been identified as knowledge gaps.

We found that the evidence-base used for emergency planning in the UK was fragmented and its robustness inconsistent as has been reported elsewhere [8,13,16]. The application of the evidence-base is also reported to be patchy [3-5], and decision-making tends to be dependent on the individual and organisational culture of the agencies involved [13]. This was clearly exemplified by the current approach of emergency planning practitioners tending to “muddle through” situations, often relying on their previous experience and intuition, rather than any robust evidence base or understanding of the wider socio-political or behavioural aspects. Indeed, past experience from previous inquiries into disasters is of lessons not learnt and mistakes being repeated [17,18]. What is therefore called for is a professional culture shift towards a much more evidence-based approach to emergency management [8]. There is a vital need to embed learning from past events and knowledge into current practice as well as identifying mechanisms that would ensure organisational memory is not lost [19,20].

Our study also reiterates current gaps in the understanding of how individuals behave in emergency situations, both at the individual as well as the community and organisational levels [21-23]. Assumptions are often made in the planning process as to how individuals will react that are insufficiently grounded in an evidence base. This may have a significant bearing on how events unfold and eventual outcomes of the emergency situation. There is also a tendency to disregard knowledge from sources outside the UK as irrelevant to the local context [13]. This view ignores a substantial body of knowledge on emergency planning from around the world, and in particular the United States [13,16]. In addition to learning from UK sources, the assimilation and synthesis of knowledge from abroad would help build up the evidence-base and address knowledge gaps.

### What this study adds

There is a risk that studies of emergency planning are carried out in detail at a microscopic level. Such deconstruction of the system to its component parts may insufficiently address many of the issues and challenges encountered in reality where the response to an emergency is by the “system” as a whole. Furthermore, the emergency management cycle (Figure 1) should be examined in its entirety and not be restricted to the response phase only [12]. Indeed, recovery and mitigation issues are frequently overlooked [13,16]. There are various issues specific to these other phases, such as psychosocial trauma, community cohesion, and the health impacts of poorly managed incidents that are often inadequately documented and studied [24].

Numerous questions remain unanswered such as the optimal configuration of the emergency management system or its approach to disaster situations: flexible or standardized, top-down or bottom-up, reactive or proactive, generic versus specific planning [21]. Does the current system enable or disable responders? Neither is it clear how the performance of the system can be measured [25,26]. For example, what would constitute “a good recovery”? It may be the case that there is no formulaic response to disasters that is guaranteed to work in all settings but what would be sought is a system that is able to apply a blend of approaches to maximise the likelihood of a good outcome.

It is also uncertain how the public in the UK socio-political setting can be meaningfully engaged in emergency management. Currently the relationship between practitioners, the emergency management system and the public appears to be less than ideal. Public involvement tends to be minimal and tokenistic and this is reinforced by an emergency management system and culture that is disempowering [2]. Furthermore, the *“failure to communicate”* with the public has been identified as a recurrent issue from emergencies and exercises [21]. It would be beneficial to better understand why this failure to communicate occurs and how it might be rectified. As evidence from elsewhere indicates, community resilience and community-based disaster risk reduction can only be achieved through meaningful engagement and empowerment of the community [24,27].

### Limitations of the study

This study was an exploration of emergency planning issues in the UK context and its transferability to other contexts may therefore be limited without appropriate interpretation. The study respondents were predominantly current practitioners in the field of emergency planning. Consequently, their views may not fully reflect the wider discourse that includes academic and political perspectives. That said, the majority of respondents were of considerable seniority within their respective organisations and were able to draw on insights not just from the operational end but also from the tactical and strategic perspectives of emergency planning.

## Conclusion

In conclusion, numerous issues have been identified where there is value in exploring further. There is a need to build a UK evidence-base founded on robust research of individual, population, organisational and system-level themes in emergency planning. This evidence needs to be translated into action and embedded into organisations with the ultimate aim of developing a health system and community that is resilient to disasters.

## Abbreviations

HPA: Health protection agency; NHS: National health service; UK: United Kingdom.

## Competing interests

The authors declare they have no competing interests.

## Authors’ contributions

All 4 authors were involved in the conceptualization and design of the study. AL and WP conducted the study. AL carried out the analysis and interpretation of the data, and drafted the manuscript. All authors reviewed the analysis and interpretation of the data, as well as the manuscript critically for important intellectual content and have approved the final manuscript.

## Pre-publication history

The pre-publication history for this paper can be accessed here:

http://www.biomedcentral.com/1471-2458/12/884/prepub
